# Individual-level predictors of inpatient childhood burn injuries: a case–control study

**DOI:** 10.1186/s12889-016-2799-1

**Published:** 2016-03-01

**Authors:** Homayoun Sadeghi-Bazargani, Reza Mohammadi, Shahrokh Amiri, Naeema Syedi, Aydin Tabrizi, Poupak Irandoost, Saeid Safiri

**Affiliations:** Road Traffic Injury Research Center, Department of Statistics & Epidemiology, Tabriz University of Medical Sciences, Tabriz, Iran; WHO Collaborating Center on Community Safety Promotion, Karolinska Institute, Stockholm, Sweden; Research Center of Psychiatry and Behavioral Sciences, Tabriz University of Medical Sciences, Tabriz, Iran; School of Pharmacy and Medical Sciences, Sansom Institute for Health Research, University of South Australia, South Australia, Australia; Child Health Research Center, Tabriz University of Medical Sciences, Tabriz, Iran; Managerial Epidemiology Research Center, Department of Public Health, School of Nursing and Midwifery, Maragheh University of Medical Sciences, Maragheh, Iran; Department of Epidemiology and Biostatistics, School of Public Health, Tehran University of Medical Sciences, Tehran, Iran

**Keywords:** Burns, Injuries, Risk factors, Child, Epidemiology, Predictors, Case- control studies, Iran

## Abstract

**Background:**

Burn injuries are considered one of the most preventable public health issue among children; however, are a cause of significant morbidity and mortality in Iran. The aim of this study was to assess individual-level predictors of severe burn injuries among children leading to hospitalization, in East Azerbaijan Province, in North-West of Iran.

**Methods:**

The study was conducted through a hospital based case–control design involving 281 burn victims and 273 hospital-based controls who were frequency matched on age, gender and urbanity. Both bivariate and multivariate methods were used to analyze the data.

**Results:**

Mean age of the participants was 40.5 months (95 % CI: 37–44) with the majority of burns occurring at ages between 2 months-13.9 years. It was demonstrated that with increase in the caregiver’s age there was a decrease in the odds of burn injuries (OR = 0.94, 95 % CI: 0.92-0.97). According to the multivariate logistic regression there were independent factors associated with burn injuries including childhood ADHD (OR = 2.82, 95 % CI: 1.68 - 4.76), child’s age (OR = 0.73, 95%CI: 0.67 - 0.80), flammability of clothing (OR = 1.60, 95 % CI: 1.12 - 2.28), daily length of watching television (OR = 1.31, 95 % CI: 1.06 - 1.61), playing outdoors (OR = 1.32, 95 % CI: 1.16 - 1.50) and increment in the economic status (OR = 1.37, 95 % CI: 1.18 - 1.60).

**Conclusion:**

Major risk predictors of burn injuries among the Iranian population included childhood ADHD, child’s age, watching television, playing outdoors, high economic status and flammable clothing.

## Background

Irrespective of the age, morbidity and mortality due to burn injuries accounts for about 12 % and 9 %, respectively with an estimate of 5 million deaths worldwide in 2000 [1, 2]. Among different types of injuries, burn injuries are severe resulting in mortality, quality of life impairment and disability. Burn injuries contribute as a major cause of morbidity and mortality among all age groups, particularly in children and adolescents living in the low and middle income countries (LMICs) [3–6]. Burn injuries are considered an important preventable cause of injuries among children; however, they still result in significant morbidity and mortality in Iranian population [7–9]. According to the National Burden of Disease Study in 2003, in Iran burn injuries are ranked as 13th most frequent cause of the burden of disease in the general population, and 7th in children aged 5–14 years [7]. In addition, according to the Tehran Forensic Medical Council, burn injuries are ranked second after traffic injuries accounting for 18 % of mortality among children [10].

Burns are treatable injuries, however, the stages of treatment are sophisticated, expensive, and time consuming since patients require special care and equipment, as well as well-trained staff. Hence it is more gratifying to prevent it rather than to treat it and therefore exploration of epidemiological characteristics of this injury is essential [7, 8].

In order to characterize the epidemiology to deliver effective prevention, it is important to have a clear understanding of the etiological patterns as there is a difference in the cultural and socioeconomic factors and the availability of health care facilities even within a country [11]. Interestingly, previous studies focused on collecting evidence from high-income countries (HICs) [12]. Although the patterns, risk factors and prevention strategies of burns can be quite different in HICs in comparison to LMICs, a few of these interventions are transferable to LMICs [13–16].

Despite the severity of burn injuries among LMICs and eastern Mediterranean countries, to date, very few studies have focused on the predictors of burn injuries [11, 17]. Moreover, minimal attention is paid to predictors of burn injuries among children. These predictors could be demographic, physical, neurological, psychological and behavioral factors such as epilepsy, attention disorders and birth order. These are reported mostly in descriptive studies and also in some case–control and cohort studies which require checking for consistency in various settings. One important potential burn injury predictor to be investigated could be attention deficit/hyperactivity disorder. Some previous cross-sectional, case–control and cohort studies have reported association of this predictor with injuries, however, its specific association with burn injuries is not well documented in literature especially when assessed along with other potential predictors of burns [11, 18–24]. The aim of this study was to assess the Individual-level predictors of inpatient childhood burn injuries in East Azerbaijan, the North-West province of Iran.

## Methods

A hospital-based, case–control study was conducted for a period of 12 months during 2009–2010 at Sina University General Hospital in Tabriz, a city in the northwest of Iran and capital of East-Azerbaijan province. This hospital is a referral burn center providing a tertiary level of care, and serves as the referral center for the nineteen districts of province with a population of around four million people. Cases and controls were frequency matched on age, gender and urbanity. The age range of all subjects in both groups lied between 6 months and 12 years.

According to the WHO definition, a burn is an injury to the skin or other organic tissue primarily caused by heat or due to radiation, radioactivity, electricity, friction or contact with chemicals. Skin injuries due to ultraviolet radiation, radioactivity, electricity or chemicals, as well as respiratory damage resulting from smoke inhalation, are also considered to be burns [25]. These fall into ICD 10 chapter 20: X00-X19 and chapter 19: T20-T32 coding categories. Exposure to electric current (W85-W87) was also included if leading to any of the injuries in T20-T32 coding categories. To serve the objectives of this study, only thermal burns will be investigated.

### Cases

This study involved 281 injured children from all social classes and both genders, who were hospitalized in the Sina University Hospital during the years 2009–2010. All inpatient burn victims with an unintentional acute thermal injury fulfilling inclusion criteria were invited to participate in the study irrespective of their later outcome, death or discharge, during the hospitalization process. That is to say those who died before the complete interview and assessments were not included and those who died after the completion of interviews were included.

### Controls

As the other wards in Sina hospital accepted adults, 273 hospital-based controls for this study were selected from another university hospital-Tabriz Pediatric Hospital, with population and referral pattern similar to Sina children’s burn injuries wards. All subjects were selected as per the control selection principals for case–control studies during the years 2009–2010 [26–28]. However, all wards were not included. For example the general pediatric clinic that received patients mostly from the nearby regions was excluded to prevent violation of the common source population principal in selecting controls for case control studies. Moreover, even at included wards, the control selection process was done on a per-case assessment. For example, children under long-term intensive treatment of diseases severely affecting the life style, such as renal dialysis, were excluded to ensure the independence of exposure from selection while minimizing the recall bias. As a distinct example, a child who is under heavy oncologic treatment for the past six months will have different rates of exposures such as playing outdoor or watching TV when compared to the ideal control population and it will also be unreliable to ask how was the situation prior to the long-term course of disease. Exclusion of these controls helped to diminish introducing the chance of either selection bias or recall bias.

All patients willing to participate in the study who had the following inclusion and exclusion criteria were enrolled as the **case** group:

Inclusion criteria:

1. Patients residing in the East Azerbaijan Province, at least for a month, either as permanent residents, passengers or other forms of residents.
2. Patients with manifestation of burn injuries that occurred either indoor or outdoor.

Exclusion criteria:

1. Burn injuries that had not taken place in East Azerbaijan Province.
2. Patients with intentional burns and self-immolation.
3. Patients with non-thermal burn injuries like chemical burns and frostbites (cold burns).
4. Patients involved in fire catastrophes and disasters.
5. Patients with burn injuries involving child abuse.
6. Outpatient admissions.
7. Patients with burn injuries with incomplete medical data.

Patients willing to participate in the study and had the following inclusion and exclusion criteria were enrolled as **control** group:

Inclusion criteria:

1. Patients living in East Azerbaijan province with no history of burn injuries a month prior to enrollment in the study.
2. Patients admitted to Tabriz Pediatric Hospital for other reasons.
3. Patients of the similar age, gender and urbanity status (rural vs urban) to the case group.

Exclusion criteria:

1. Patients suffering from chronic diseases or other major types of injuries.
2. Outpatient admissions.

### Variables tested/assessed in this study

Hospital medical records were utilized to retrieve information regarding injury outcome, severity, extent of burn, and ICD 10 coding. Validity of the information collected was increased by initially collecting primary data via questionnaire followed by the last stage in data collection obtained from ICD coding. The questionnaires were completed by the interviewer during the interview process with some exceptions where individuals filled in their own questionnaire, which was later reviewed by the interviewer. For the purpose of this study 4 interviewers were chosen from the hospital’s medical registry staff including three medical registry experts. These staff had completed a two-year long academic education in the medical registry before being employed by the hospital. In addition to this they participated in a short training session and conducted a supervised pilot data collection to ensure lower interviewer variability. The reliability and validity of data collection was confirmed by getting each interviewer to conduct same number of interviews with cases and the matched controls. This is to diminish the information bias through the comparable accuracy principal in case–control studies. As this study involves assessing burn injuries of children, either their parents or caregivers were interviewed.

Furthermore, other variables assessed in this study included demographics, factors relating to children, caregivers and socioeconomic elements. As the flammability standards for children clothes are not available in Iran, it was measured using a likert scale assessing for the two factors of looseness and specific fabric synthetics content of the clothing. Childhood Attention Deficit Hyperactivity Disorder was assessed using the ADHD-Rating Scale [29]. Total score of ADHD was computed prior to its use in the adjusted models, due to collinearity between the subscales of ADHD. ADHD ratio was calculated per ten score increment for a better understanding.

Since this study involves assessing economic status of a LMIC, it was ideal to use consumption expenditure method. In order to use this method via Principal Component Analysis, economic status composed of a single variable on which weighted aggregation of a collection of cost expressing variables is done. The single variable was then transformed into quintiles.

The variables measured in the present study included; ability to provide clothing, food, Jewelry, furniture, traveling and Education costs.

All stages of analysis involve determining the suitable scales of variables and model building as per recommendations by Jewell in Statistics For Epidemiology [30].

### Statistical analysis and sample size

The variables used in this study were analyzed using both bivariate and multivariate methods. Statistical analyses were done using Stata statistical software package (Release 9. College station, TX: StataCorp LP.). An independent samples *t*-test was used to compare the means of normally distributed numeric independent variables for both control and case groups. The Mann–Whitney *U* test was used as a nonparametric analog to the independent samples *t*-test when the normality assumption didn’t hold and in such conditions median was reported. In order to assess the association of two categorical variables, Chi-square test was applied. Crude odds ratios were calculated and their 95 % confidence intervals were reported. Variables with associations with a *p*-value < 0.1 were adjusted in multivariate conditional logistic regression analysis. The adjusted odds ratios along with their 95 % confidence intervals were kept in the final model. In this study statistical significance was set with a *p*-value < 0.05 (two tailed test). A sample size of 283 per group was calculated in order to detect at least a 6 % difference in proportions of the exposure from the baseline 4 % proportion and to fulfill a minimum statistical power of 80 % and 95 % confidence level.

### Ethical issues

All protocols were approved by regional ethics committee of Tabriz University of Medical Sciences. Research was carried out in compliance with the Helsinki Declaration. Data from burn injuries was only collected with informed consent of parents and complementary assent of children higher than 7 years old.

## Results

**Fig. 1:**
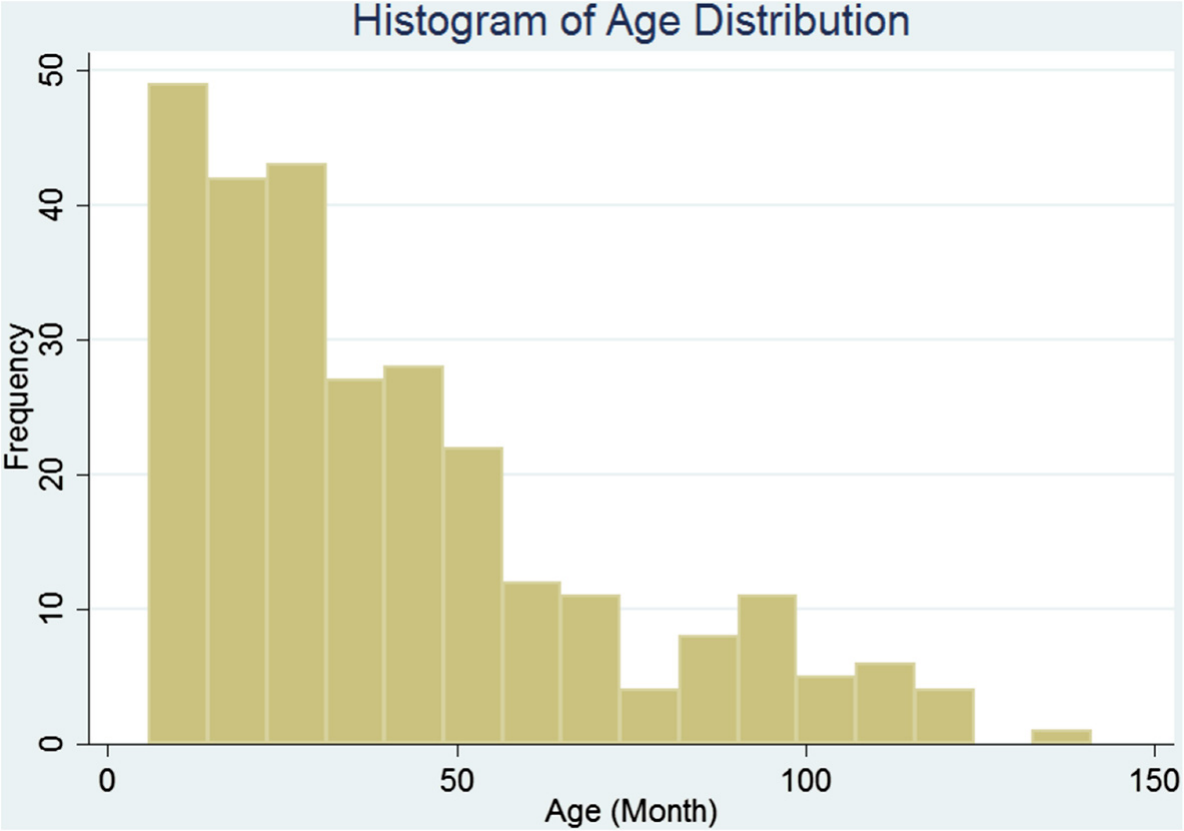
Age distribution of burn victims in an Iranian children population

Males comprised 57 % of the participants of the study. The mean ± SD of age was 40.5 ± 1.7 months (ranging: 6–144 months). About 70.1 % of participants with burn injuries were under four years of age (Fig. 1). About 84 % of injuries occurred at home (indoors) with 44 % and 25 % of them taking place in kitchen and rooms, respectively and about 47 % of burns were scalds. It was found that majority of caregivers of the participants involved in this study were females (99 %). For further details comparing case and control groups refer to Table 1.

**Table 1:** Comparing the baseline characteristics between the burned children and controls

Variables	Case (*n* = 281)	Control (*n* = 273)	*p*-value^c^
Age (year)^a^	3.27 (0.14)	5.66 (0.23)	<0.001^c^
Sex (male)^b^	170 (61)	143 (53)	0.06
Birth order	1.76 (0.96)	1.88 (1.15)	0.2
Family size	4.64 (2.56)	4.69 (1.86)	0.8
Total ADHD			
Inattentive	2.89 (0.17)	2.30 (0.15)	0.01^c^
Hyperactive	7.91 (0.30)	6.51 (0.30)	0.001^c^
Economic status			0.001^c^
SES (richest)	42 (16.5)	74 (28.4)	
SES (second richest)	43 (16.9)	48 (18.4)	
SES (middle)	50 (19.7)	59 (22.6)	
SES (second poorest)	59 (23.2)	38 (14.6)	
SES (poorest)	60 (23.6)	42 (16.1)	
Watching TV (hours)	1.78 (1.63)	2.36 (1.77)	<0.001^c^
Playing out of the house (hours)	2.03 (2.29)	1.25 (1.69)	<0.001^c^
Level of flammability of cloths			0.003^c^
Very low	21 (7.6)	31 (11.4)	
Low	135 (48.9)	159 (58.5)	
High	116 (42)	82 (30.1)	
Very High	4 (1.4)	0 (0)	
Having burns history in last year (yes)	12 (4.29)	18 (6.64)	0.2
Having burns history in family (yes)	68 (24.37)	72 (26.47)	0.6
Having burns scar (yes)	70 (25.83)	48 (17.71)	0.02^c^
Having epilepsy (yes)	9 (3.25)	18 (6.64)	0.07
Having musculoskeletal disorders (yes)	9 (3.22)	13 (4.81)	0.3

^a^Variables with numeric scales are reported as Mean (standard deviation)

^b^Variables with categorical scales are reported as Number (percent) ^c^p-value ≤0.05

### Bivariate analysis results

The proportion of males was 61 % in case group versus 53 % among controls but with a borderline *P*-value (Chi-square = 3.55; P =0.059). The median household size was 4 (i.e. number of household members) in both the case and control groups with no statistical significance (Mann–Whitney *U* test: *P* = 0.1). The proportion of subjects with musculoskeletal disorder in case and control groups were 3.3 % and 5 % respectively, however it was not statistically significant (Chi-square = 0.9; *P* = 0.343).

Participants with epilepsy were not significantly different from others in burn likelihood (OR = 0.47, 95 % CI: 0.2-1.07). Similarly participants with history of their and of one of their family member’s burn injuries in the past year (OR = 0.62, 95 % CI: 0.29-1.33) and (OR = 0.89, 95 % CI: 0.61-1.31) respectively. Additionally the odds of burn injuries were increased by 80 % for those whose caregivers were not mothers; however this was statistically not significant (OR = 1.80, 95 % CI: 0.65-4.95).

There was an association between age of children and burn injuries where the odds of burns decreased by 20 % with an increase in the child’s age (OR = 0.80, 95 % CI: 0.75 - 0.84). In addition to this, there was a significant decrease (19 %) in the chance of burn injuries with one hour increment in watching television (OR = 0.82, 95 % CI: 0.74-0.91). In contrast to this, watching television with one hour outdoor activities, significantly increased the odds of burn injuries by 21 % (OR = 1.21, 95 % CI: 1.10-1.33). Moreover, there was a significant increase in the odds of burn injuries with an increase in level of flammability of cloths (OR = 1.59, 95 % CI: 1.21-2.08).

There was a significant decrease by 6 % in the odds of burn injuries with one year increment in the caregiver’s age and burn injuries (OR = 0.94, 95 % CI: 0.92-0.97).

It was shown that, children with higher economic status had high chance of burn injuries, such that, with one unit increase in economic status, the odds were increased by 27 % (OR = 1.27, 95 % CI: 1.12-1.44).

**Fig. 2:**
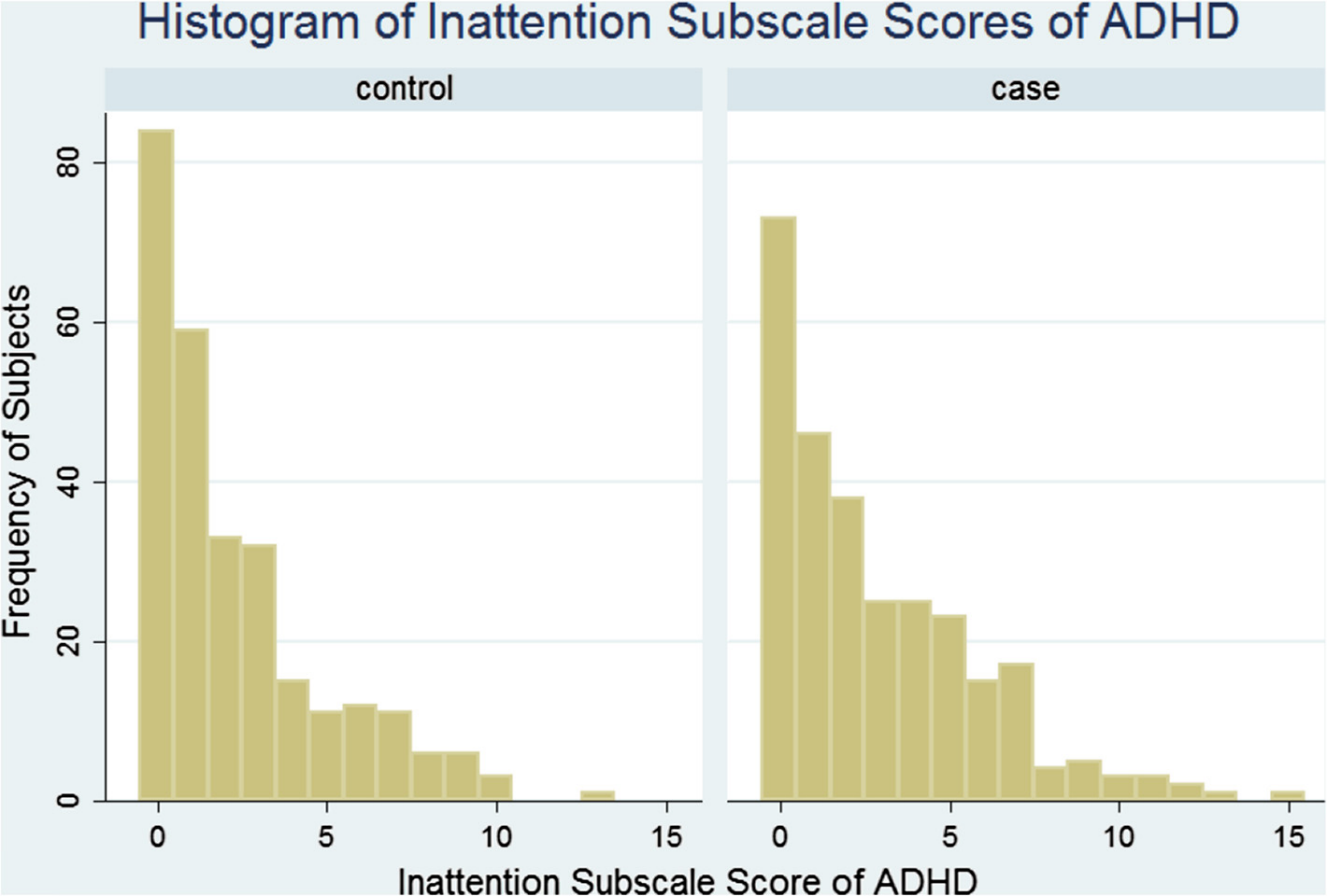
Histogram of the distribution of ADHD Inattention subscale among the burned cases and matched controls

**Fig. 3:**
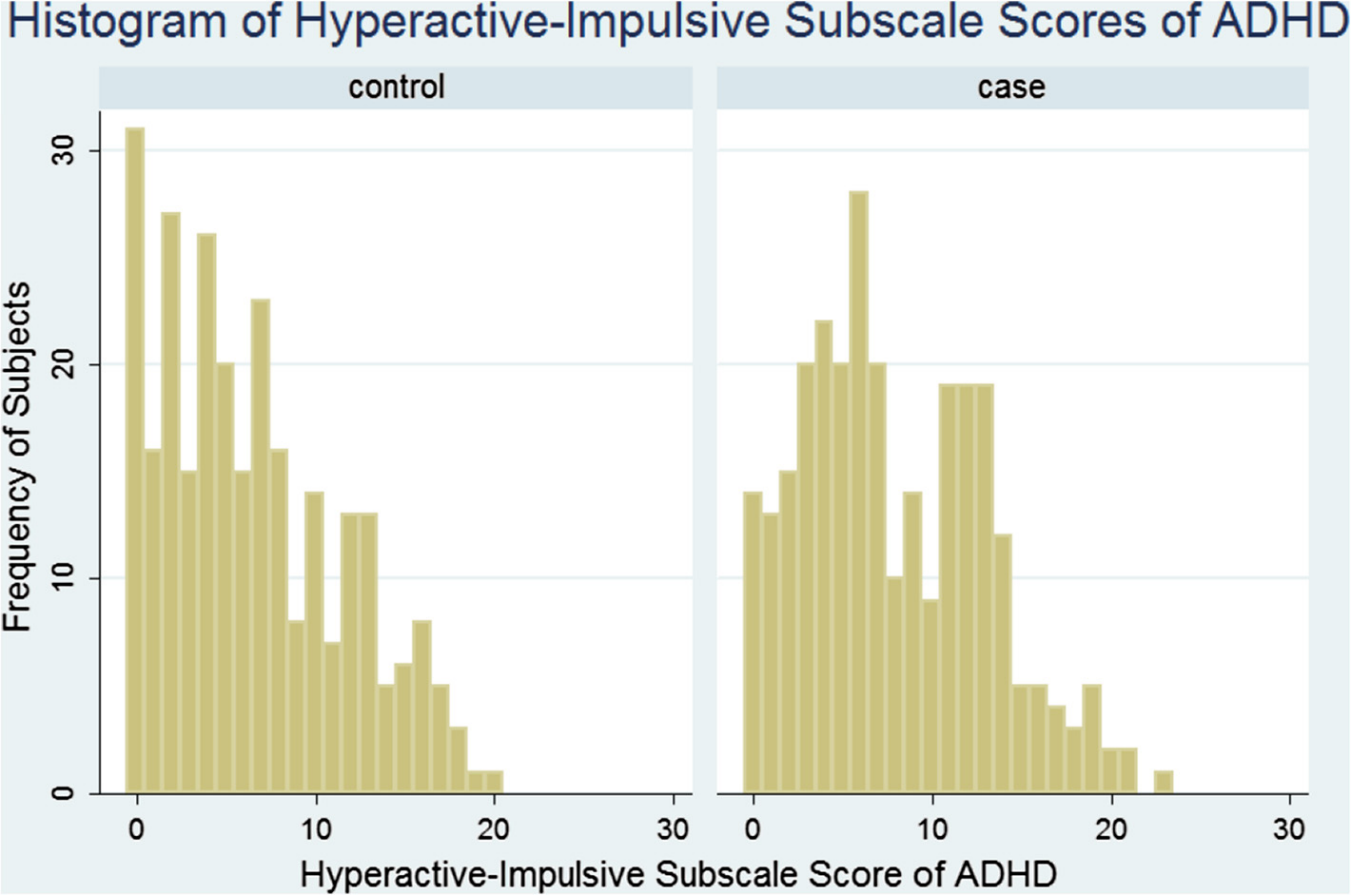
Histogram of the distribution of ADHD Hyperactive-Impulsive subscale among the burned cases and matched controls

It was evident that distribution of Childhood ADHD varied between the two study groups with high scores prevalent among the case group (Figs. 2 and 3). Children with higher level of inattention significantly had the higher odds of burn injuries, such that, with ten units increment in the score of inattention subscale the chance of burn injury was increased by 115 % (OR = 2.15, 95 % CI: 1.16-3.96). Furthermore, it was shown that with ten units increment in the score of Hyperactive-Impulsive subscale of ADHD, 73 % increment was observed in the chance of burn injuries (OR = 1.73, 95 % CI: 1.24-2.42). There was significant interaction between Childhood ADHD and watching television on burn injuries, such that, with one unit increases in watching television, the association between childhood ADHD and burn injuries decreases (Table 2).

**Table 2:** Crude and adjusted odds ratios for predictors of childhood burn injuries included in the final logistic regression model

Predictors in model	Crude odds ratio (95 % CI)	Adjusted odds ratio (95 % CI)
Child’s age	0.80 (0.75–0.84)	0.73 (0.67–0.80)
Watching TV	0.82 (0.74–0.91)	1.31 (1.06–1.61)
Playing out of the house	1.22 (1.11–1.33)	1.32 (1.16–1.50)
Level of flammability of cloths	1.59 (1.21–2.08)	1.60 (1.12–2.28)
Economic status	1.27 (1.12–1.44)	1.37 (1.18–1.60)
Childhood ADHD	1.49 (1.17–1.90)	2.82 (1.68–4.76)
Interaction between Childhood ADHD and Watching TV	0.78 (0.67–0.91)	0.76 (0.63–0.91)

*ADHD* Attention deficit hyperactivity disorder

For ADHD rating scale, odds ratio was calculated per 10 score increment in scale scores

### Multivariate analysis results

The multivariate analysis consisted of six variables in the final model. According to the multivariate logistic regression results following variables were considered to be independent factors associated with childhood burn injuries: Childhood ADHD, age of child, level of flammability of cloths, watching television, playing outdoors and economic status. Similar to the bivariate analysis, interaction between Childhood ADHD and watching television was statistically significant. Despite the crude analysis, caregiver’s age was not significantly associated with childhood burn injuries after adjusting age of children and other factors in the final model (OR = 0.99, 95 % CI: 0.96-1.03) (Table 2).

## Discussion

It was evident from this study that there is a significant association between childhood burn injuries and various variables including childhood ADHD, flammability of clothes, watching television, outdoor activity, caregiver’s and child’s age, and economic status. All of these variables except caregiver’s age were independently associated with childhood burn injuries.

The present study also determined that majority of burn victims were between 1–4 years old. Higher number of burn injuries among infants and toddlers was largely attributed to their total dependence on parents and caregivers. These findings were supported by previous studies [10, 31, 32]. Moreover, findings of the current study consistent with the literature suggests that flammable clothing plays a vital role in initiating or aggravating the fire and was proven to be significantly associated with higher number of severe burn injuries [33]. Another previous study reported that death among 40 % and 30 % of burn victims is due to wearing synthetic and semi-synthetic garments respectively [34].

According to the initial analysis, it was found that there was a significant negative association between watching television and childhood injuries; however after adjustment of age and other variables, the association between the watching television and childhood injuries transformed to a significant positive association. This change in the direction of association may indicate the existence of qualitative confounders. It may be possible that the role of watching television on childhood injuries can be attributed to age of a child. For instance, older children are more likely to watch television hence, leading to lower odds of burn injuries [10, 32].

According to the present study, regardless of gender and other factors, playing outdoors was associated with burn injury, possibly due to minimal parental control during outdoor activities. Interestingly, many studies reported outdoor activities as a higher risk factor for burn injuries even among adults [35, 36]. However, to date, implications of outdoor activities among children were not discussed clearly in the literature.

In addition to above, it was evident that caregiver’s age and burn injuries are not associated independently. A possible explanation could be that, older children have older caregivers or parents whereas younger children may have younger caregivers or parents. Hence there is a decrease in the odds of burn injuries among the older group which can be attributed to children being older. Previous studies have found significant negative association between caregiver’s age and burn injuries in children [37, 38], even after adjusting for age of children [37] and other variables (e.g. child birth weight) [38]. A study done by Libber et al. in 1984 supported our findings and found that the maternal age of children (aged 15 years and under) with burns does not differ significantly from that of mothers of children without burns [39].

According to this study, there is an independent significant positive association between economic status and childhood burn injuries. Children with high economic status households were found to be more prone to burn injuries. This finding was partly supported by previous studies with controversial results. For instance, some studies state that children with moderate economic status had higher odds of injuries than low economic status children [40] whereas other studies found children with a low economic status were more likely to suffer from burn injuries [41, 42]. A possible explanation for these discordant findings regarding economic status and the childhood burn injuries is the difference in methods used for assessing the economic status. Certain methods of data collection of socio-economic factors include consumption expenditure data. This method may generate incorrect reports due to in-accuracy in recalling details or even un-willingness to accurately disclose certain types of consumption expenditure [43]. Furthermore, studying economic status as a single measure without taking into account its potential interactions and associations with other components of the socioeconomic status may sometimes be misleading. Secondly, the expenditure capacity assessed in this study may not essentially be the same as economic status. At the time this study was started, no validated Iranian socioeconomic status assessment tool was available and the researchers used their own version of assessing socioeconomic status. Recently an Iranian Socioeconomic Status assessment tool (SES-Iran) is being presented which is validated on a large population sample on two megacities (Tehran & Tabriz) as well as many other districts in Iran [44, 45]. Further studies in the future using this tool will be beneficial.

Irrespective of the well-known environmental, appliance or SES-related risk factors of the burns, current study confirmed that there exist some individual predictors of burns including; age, gender, birth order, type of clothing worn, personal activity patterns such as (sleeping and playing), child activity score and child attention deficit/hyperactivity disorder along with other individual predictors from literature such as ignorance by parents and being a migrant offspring [23, 24, 41, 46–51]. ADHD is shown to be associated with injuries regardless of the type of injury mechanism [52–55]. A most specific individual predictor of burns or a severity predictor could be the childhood ADHD. This has been investigated in several studies either specifically assessing ADHD or the activity scores [18, 19, 24, 46, 56–58]. The plausibility of the association of ADHD and burn injuries among children could be explained in several ways as follows; hyperactive children may have increased risk of hitting hot material while moving around or crashing people carrying hot material; the attention deficit problem in children with ADHD may lead to careless handling issues related to burn hazards; the higher impulsivity observed among children with ADHD is another issue increasing the risk of both intentional and accidental burns; the risk taking behaviors of children with ADHD is another explanation sometimes referred to as sensation seeking behaviors; Finally, as ADHD has a familial aggregation it should also be taken into account that children with ADHD are more likely to have parents with ADHD who in turn may be less attentive care givers to provide a safe environment for their children. It has been shown that the parents of children with ADHD have higher proportions of inattention/cognitive problems, hyperactivity/restlessness, impulsivity/emotional liability, and lower self-concept than parents of children without ADHD [59]. ADHD is a rather prevalent disorder associated with physiological disorientations in frontal brain circulation [60, 61]. The value of screening for ADHD in prevention of burn injuries may be a focus of future research considering the feasibility in screening, diagnosis and treatment of ADHD. Moreover, parents of the children with ADHD should be advised to improve their home safety as well as sticking to medical treatment given to their child. In addition to this, our study also found that there is no interaction between child’s age and ADHD on burns, however, a study in the past, found that there is a stronger association between ADHD and burn injuries among children less than four years old [62]. On the other hand, negative interaction was observed between watching television and ADHD on burn injuries; such that, with increasing the time of television watching, the association between childhood ADHD and burn injury would be reduced. It can be interpreted that with increasing television time, inattentive and hyperactive activities may decrease leading to reduction in the incidence of burn injuries.

This study was conducted on a hospital based selection of cases and controls. As discussed by Wacholder [26], case–control studies conducted on a primary study base (such as in population based selection of cases and controls) versus a secondary base (such as selecting from hospitals) have their own advantages and disadvantages. Primary study base case–control studies are encountered with a major challenge of complete case identification as well as the practical difficulties which in turn may even affect the validity. At the same time the major challenge with secondary base studies like the current one, is the definition of the study base. In secondary base studies it is quite vital to take care of ensuring common source population at selecting the controls and this was the reason for excluding the wards or hospitals with a smaller referral coverage of just the capital or the regions close to the hospital in our study.

Other than the limitations in assessing the socioeconomic status, as discussed earlier, one Flimitation in the present study was that minor burns had much lower likelihood of being enrolled in this study hence limiting the extrapolation of results for such burns. Although, severe burns have higher importance in injury prevention programs, future research is recommended to specifically determine risk factors of minor and moderate burns or comparing their etiologic pattern in comparison to severe burns [5]. It is important to note that some risk factors may be different among the three types of thermal burns (contact burns, flame burns and scalds). Certain factors such as flammability of clothing could be more precisely assessed when the study is conducted for a specific type of burn injury. However, similar to the previous studies, the purpose of this study was to assess the risk factors of burns as a whole. Future studies are recommended to investigate whether risk factors of the burns or their magnitude may vary according to the type of thermal burn injury mechanism. The tradeoff between conducting age specific versus wide age group case–control studies or the restriction to a given age group should also be thought of in small or medium sized studies.

A limitation of this study was that, in spite of a moderate sample size and two-year long census enrollment, the study was not large enough either to do subgroup analysis for the outcome; such as for scalds, flame, and contact burns; or to do subgroup analysis for the predictors such as for gender and age groups. Nevertheless, it doesn’t seem to jeopardize the main objective of study and provides better generalizability for the whole population and general prevention programs. The main strength of this study was that a wide range of possible burn injury predictors were measured and properly addressed.

## Conclusions

It was evident from this study that childhood ADHD, age of children, watching television, outdoor activities, high economic status and flammable clothing are risk predictors of burn injuries in this population and should be considered in the preventive measures.

## Abbreviations

ADHD: attention deficit hyperactivity disorder
CI: confidence interval
LMICs: lower middle income countries
OR: odds ratio
